# *TP53* Status is Associated with Thrombospondin1 Expression *In vitro*

**DOI:** 10.3389/fonc.2013.00269

**Published:** 2013-10-29

**Authors:** Angeles Alvarez Secord, Marcus Q. Bernardini, Gloria Broadwater, Lisa A. Grace, Zhiqing Huang, Tsukasa Baba, Eiji Kondoh, Gregory Sfakianos, Laura J. Havrilesky, Susan K. Murphy

**Affiliations:** ^1^Department of Obstetrics and Gynecology, Division of Gynecologic Oncology, Duke Cancer Institute, Duke University Medical Center, Durham, NC, USA; ^2^Biostatistics, Cancer Center Biostatistics, Duke Cancer Institute, Duke University Medical Center, Durham, NC, USA; ^3^Department of Gynecology and Obstetrics, Graduate School of Medicine, Kyoto University, Kyoto, Japan

**Keywords:** *Thrombospondin1*, ovarian carcinoma, methylation, *TP53*, angiogenesis

## Abstract

**Objectives:** To elucidate the association between thrombospondin1 (THBS1) expression and *TP53* status and *THBS1* promoter methylation in epithelial ovarian cancer (EOC).

**Methods:** Epithelial ovarian cancer cell lines with known *TP53* status were analyzed for *THBS1* gene expression using Affymetrix U133 microarrays and promoter methylation by pyrosequencing. *THBS1* mRNA expression was obtained pre- and post-exposure to radiation and hypoxia treatment in A2780 parent wild-type (wt) and mutant (m)*TP53* cells. *THBS1* expression was compared to tumor growth properties.

**Results:** *THBS1* gene expression was higher in cells containing a wt*TP53* gene or null *TP53* mutation (*p* = 0.005) and low or absent p53 protein expression (*p* = 0.008) compared to those harboring a missense *TP53* gene mutation and exhibiting high p53 protein expression. Following exposure to radiation, there was a 3.4-fold increase in *THBS1* mRNA levels in the m*TP53* versus wt*TP53* A2780 cells. After exposure to hypoxia, *THBS1* mRNA levels increased approximately fourfold in both wt*TP53* and m*TP53* A2780 cells. Promoter methylation levels were low (median = 8.6%; range = 3.5–88.8%). There was a non-significant inverse correlation between *THBS1* methylation and transcript levels. There was no association between *THBS1* expression and population doubling time, invasive capacity, or anchorage-independent growth.

**Conclusion:** *THBS1* expression may be regulated via the *TP53* pathway, and induced by hypoxic tumor microenvironment conditions. Overall low levels of *THBS1* promoter methylation imply that methylation is not the primary driver of *THBS1* expression in EOC.

## Introduction

Thrombospondin1 (THBS1) is a potent modulator of angiogenesis that has been shown to have both stimulatory (1–6) and inhibitory effects (7, 8) on the process of tumor neovascularization, proliferation, invasiveness, and progression. We previously demonstrated that THBS1 protein expression was associated with clinical outcome in women with advanced epithelial ovarian cancer (EOC) who were treated with taxane and platinum-based chemotherapy regimens (9). Specifically, women whose cancers had high compared to low THBS1 protein expression had worse progression-free (PFS) and overall survival (OS). THBS1 was shown to provide independent prognostic value after adjusting for clinical characteristics and p53 overexpression. Moreover, exploratory adjusted Cox regression modeling revealed that women whose cancers overexpressed p53 protein, which reflects the presence of missense *TP53* mutations, and expressed high THBS1 had a threefold elevation in the risk of disease progression and death compared with women whose cancers didn’t overexpress p53 or those that overexpressed p53 and expressed low THBS1 (9).

The TP53 tumor-suppressor pathway has been implicated in the regulation of *THBS1* gene and protein expression (1, 9). Dameron and colleagues demonstrated that *TP53* positively regulated *THBS1* promoter sequences and induced endogenous *THBS1* gene expression in fibroblasts (1). The exact regulatory mechanism is unknown, however, the *TP53* gene has numerous functions including transcription factor, cell cycle arrest activation, apoptosis, DNA damage repair, and protein–protein interactions. In addition, *TP53* has been shown to regulate other angiogenic factors via promoter methylation (10). The methylation of promoter-associated CpG islands has been linked to the transcriptional activity of multiple genes involved in carcinogenesis (11). Oshiro et al. proposed that wild-type (wt) *TP53* DNA-binding activity to promoters prevents aberrant methylation (10). Upon mutation the wt p53 DNA-binding activity is lost and the TP53 target regions are vulnerable to *de novo* cytosine methylation. *TP53* mutations are present in over 90% of high-grade serous ovarian cancers (12) and represent a plausible mechanism of controlling epigenetic regulation of gene transcription.

The objective of the present study was to evaluate the relationships between THBS1 protein expression, *TP53* status, and *THBS1* promoter methylation in EOC cell lines. We also sought to determine if induced *TP53* transcription or hypoxia was associated with increased *THBS1* mRNA transcription. Our primary hypothesis was that inactivation of the *TP53* tumor-suppressor gene pathway modulates *THBS1* transcription and expression in ovarian cancers through aberrant promoter hypermethylation. Furthermore, we evaluated whether *THBS1* expression was associated with population doubling time, invasive capacity, anchorage-independent growth as well as cisplatin and paclitaxel induced growth inhibition.

## Materials and Methods

### Ovarian cancer cell lines

#### Cell culture

Twenty-one immortalized ovarian cancer cell lines were included. Nineteen ovarian cancer cell lines were evaluated for *THBS1* expression and promoter methylation status, while two A2780 ovarian cancer cell lines (wt parent and mutant *TP53* daughter lines) were used to evaluate the effect of radiation and hypoxia treatment on *THBS1* mRNA expression. The cells were grown in monolayer culture in RPMI1640 media (Sigma-Aldrich Co., St. Louis, MO, USA) supplemented with penicillin and streptomycin (100 U/mL penicillin, 100 μg/mL streptomycin; Invitrogen, Carlsbad, CA, USA) and 10% heat inactivated fetal bovine serum (v/v; Invitrogen) in an atmosphere of 5% CO_2_ at 37°C.

The short tandem repeat (STR) genotypes of all ovarian cancer cell lines were analyzed to authenticate the cell lines using the AmpFLSTR^®^ Identifiler^®^ Plus PCR Amplification Kit (Applied Biosystems, Carlsbad, CA, USA) at the University of Colorado Cancer Center, DNA Sequencing, and Analysis Core (13). The STR genotypes of ovarian cancer cell lines that are available from the American Type Culture Collection or the RIKEN BioResource Center Cell Bank were identical to the source genotypes as reported within their respective STR databases and all other non-commercially available cell lines were shown to be derived from females with unique genotypes.

Protein extractions were performed as previously described (14) and RNA extractions were performed using the RNeasy Mini Kit following the manufacturer’s protocol (Qiagen, Inc.; Valencia, CA, USA). For cDNA synthesis, 1 μg of total RNA was incubated for 60 min at 42°C with oligo (dT) primers and 20 units of AMV reverse transcriptase in 1× reverse transcriptase buffer supplemented with 5 mM of MgCl_2_, 1 mM of each dNTP, and 25 units of RNase inhibitor in a final volume of 20 μl (Roche Diagnostics Cooperation, Indianapolis, IN, USA). Methodologies for determining *TP53* mutation status and immunohistochemical protein expression, population doubling time of the cells, invasive capacity, and chemotherapy-induced growth inhibition have been previously described (15–18). Western blot analysis of TP53 protein was also performed before and after exposure to radiation and hypoxia. Ten micrograms of total cellular protein for each specimen were separated by 7.5% SDS polyacrylamide gel electrophoresis and transferred to a nitrocellulose membrane (Schleicher and Schuell). Membranes were first incubated with primary antibodies against TP53 (1:3000 DO-1, mouse monoclonal, Santa Cruz Biotechnology, Inc., Santa Cruz, CA, USA) overnight at 4°C or β-actin (1:3000) A4700, mouse monoclonal, SIGMA, St. Louis, MO, USA) for 1.5 h at room temperature, and then with an anti-mouse secondary antibody (1:7500 115-035-062, Jackson ImmunoResearch, West Grove, PA, USA) for 1 h at room temperature. Antibody interactions were visualized using chemiluminescence (Perkin Elmer Western Lightning™ Chemiluminescence ECL Reagent, Shelton, CT, USA). TP53 and β-actin expression were quantified by densitometric scanning using Scion Image software (Scion Corporation, Frederick, MD, USA). Results were then normalized to the β-actin content in each lane to correct for relative expression.

#### Anchorage-independent growth

Assays for colony formation in soft agar were performed as described. (19) Briefly, 2× RPMI media was prepared from powder and supplemented with fetal bovine serum and antibiotics (Invitrogen; Carlsbad, CA, USA), and 1% agarose was made with the RPMI media using low-melting-temperature agarose (Invitrogen). One milliliter of 0.5% agarose was placed into each well of six-well tissue culture dishes and overlayed with 1 mL of 0.33% agarose prepared in 1× RPMI and containing 2 × 10^4^ cells. After 3 weeks incubation at 37°C in a humidified chamber with 5% atmospheric CO_2_, colonies larger than 100 μm in diameter were counted. The colony number formed for each cell line was determined by averaging the number of colonies >100 μm that were counted in 10−20 microscopic fields at 100× magnification.

#### Hypoxia treatment of cell lines

A2780 cell lines were grown to 80% confluence in T150 flasks and exposed to hypoxic conditions using 0.5% O_2_ 0.5% O_2_ 0.5% O_2_ 0.5% O_2_ 0.5% O_2_ in a Bactron Anaerobic Chamber (Sheldon Manufacturing: Cornelius, OH, USA) for 8 or 24 h prior to harvesting through trypsinization.

#### Radiation treatment of cell lines

Ionizing radiation was used to stimulate *TP53* induction (20). A2780 cell lines were plated in 60 mm dishes, grown to 80% confluence, and exposed to 5 Gy of ionizing radiation using the Gamma cell 1000 (MDS Nordion ON, Canada), and harvested at 0, 2, 4, 6, 8, 24, and 48 h post-exposure. To validate our model we irradiated the A2780 wild-type *TP53* (A2780wt*TP53*) ovarian cancer cell line to 5 Gy and then subjected cell lysates to immunoblot to assess p53 protein expression.

#### *THBS1* mRNA and protein expression

The genomic array and Western blot analysis were performed as previously described (9, 21) (NCBI Accession Series GSE25428; www.ncbi.nlm.nih.gov/geo). Three probes (201108_s_at, 201109_s_at, and 201110_s_at) on the Affymetrix U133 chip were used to assay the cell lines for *THBS1* expression in 19 cell lines. Expression levels were RMA-normalized, and the average expression probe value was calculated.

Real-time quantitative PCR (RQ-PCR) was used to analyze mRNA expression in eight immortalized ovarian cancer cell lines (OVCA429, OVCA433 DOV13, OVCAR3, OVCA432, SKOV3, A2780wt*TP53*, and A2780 mutant *TP53* (A2780m*TP53*). Quantification of *THBS1* mRNA expression was obtained by RQ-PCR using fluorescent TaqMan methodology (ABI Prism 7900HT Sequence Detection System; Applied BioSystems; Foster City, CA, USA). RQ-PCR was performed using 7.25 μl 1:15 dilute cDNA, 12.5 μl Taqman Universal PCR Master Mix (Applied Biosystem; Foster City, CA, USA), 1.25 μl primer assay in a final volume of 25 μl. Primers and probes for *THBS1* (Hs00170236_m1) and *GAPDH* (human 402869) were obtained from Applied Biosystems (Foster City, CA, USA). The thermal cycling conditions were: 50°C for 2 min and 95°C for 10 min followed by 50 cycles of 95°C for 15 s and 60°C for 1 min. The comparative cycle threshold method was used to calculate the relative expression of *THBS1* mRNA normalized to *GAPDH* run in parallel (22).

Thrombospondin1 protein expression was quantified by densitometric scanning (Scion Corporation, Frederick, MD, USA) and normalized to the β-actin content to correct for relative expression.

#### Methylation analyses

Bisulfite pyrosequencing was used to evaluate *THBS1* promoter methylation status in the cell lines on a PyroMark Q96 MD pyrosequencing instrument (Qiagen; Valencia, CA, USA). Genomic DNA (800 ng) was modified with sodium bisulfite as previously described (23) to convert unmethylated cytosines to uracils. Methylated cytosines are protected from this conversion. PCR amplification prior to pyrosequencing was performed with the HotStar Taq PCR Kit (Qiagen) using 40 ng of bisulfite modified DNA (assuming complete recovery) in a 25 μl reaction volume with 1.5 mM MgCl_2_ and 100 nM each of forward primer 5′-AGT TTT TTT TAG GGA TGT TTT GTT GAT-3′ and reverse primer 5′-(biotin)-CCA AAC TTA AAA ACA CTA AAA CTT CTC A-3′. PCR conditions were 95°C for 15 min, followed by touchdown PCR using 55 total cycles with a 30 s denaturation at 94°C, a 30 s annealing step (5 cycles at 69°C, 5 cycles at 66°C, 5 cycles at 63°C, 5 cycles at 60°C, and 40 cycles at 57°C) and a 30 s extension step at 72°C, followed by a final 10 min extension step at 72°C. The extended cycle number is required to fully incorporate the biotin-tagged primer, which is used to isolate the single stranded amplicon used as the template for the pyrosequencing reaction, which was done with sequencing primer 5′-GGG ATG TTT GTT GAT TAT-3′. Pyrosequencing was performed using PyroMark Gold Q96 reagents (Qiagen) per the manufacturer’s recommendations. The mean methylation value for the seven CpGs within the sequenced region was used for analysis. Low methylation was arbitrarily defined as <15% methylated, and high methylation as >15%. The pyrosequencing assay was validated using mixtures of bisulfite modified universally methylated DNA (CpGenome; Promega; Madison, WI, USA) and normal leukocyte DNA with the methylated DNA comprising 0, 20, 40, 60, 80, and 100% of the total input. There was a linear relationship between the amount of methylated DNA present in the reactions and that measured by pyrosequencing, with a correlation coefficient of 0.98.

#### Statistical analysis

Spearman’s correlation coefficient test was used to assess the association between *THBS1* gene expression and population doubling time of the cells; invasive capacity; anchorage-independent growth index; as well as cisplatin and paclitaxel IC50 values. The Wilcoxon rank sum test was used to compare the continuous representation of *THBS1* gene expression and promoter methylation in various groups defined by *TP53* gene mutation and protein expression. All tests were two-sided and *p* < 0.05 was considered statistically significant.

## Results

### *THBS1* expression and promoter methylation status in ovarian cancer cell lines

**Figure 1:**
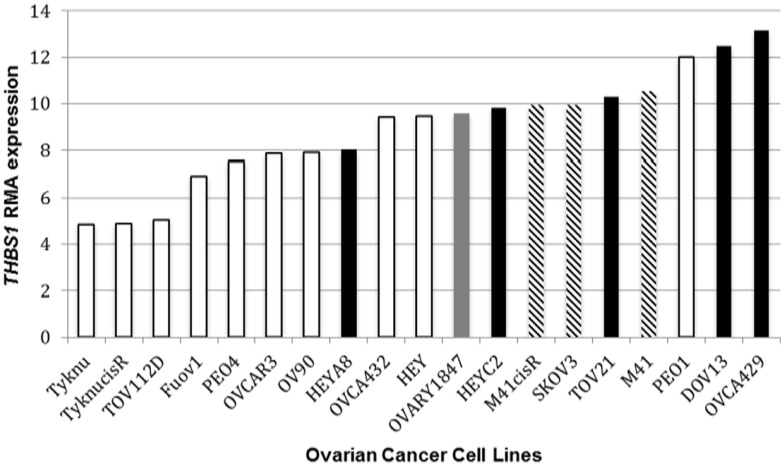
***THBS1* RMA expression and *TP53* status**. Ovarian cancer cell lines harboring a *TP53* missense mutation bars had lower *THBS1* RMA expression compared to cell lines with an intact wild-type (wt) *TP53* gene. *TP53* missense mutation 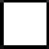; *TP53* frameshift mutation 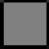; null mutation 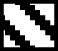; wt *TP53* gene 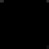. Expression values are given as log2-transformed RMA-normalized values from the Affymetrix U133A gene chip.

**Figure 2:**
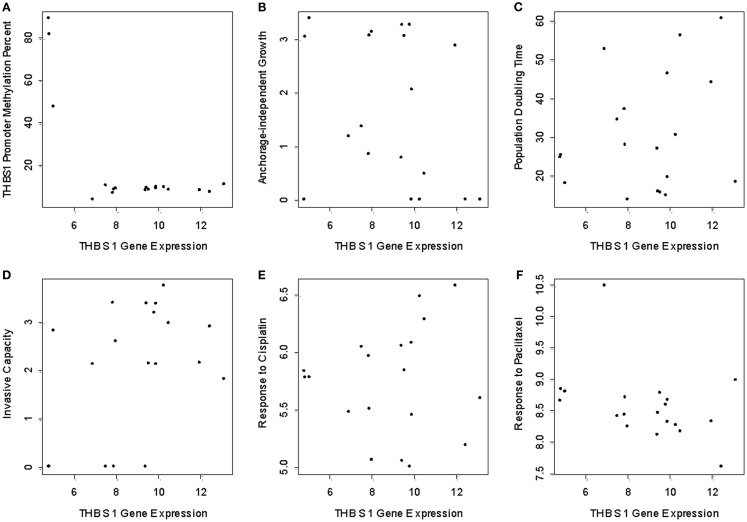
***THBS1* gene expression, *THBS1* promoter methylation, tumor growth properties, and chemotherapy-induced growth inhibition**. Pyrosequencing shows low levels of methylation in the cell lines (median = 8.6%; range = 3.5 to 88.8%). **(A)** Cells with low levels of promoter methylation exhibited higher *THBS1* gene expression, while those with high levels of promoter methylation had low *THBS1* gene expression. There was no association between *THBS1* gene expression with anchorage-independent growth; **(B)** population doubling time **(C)** invasive capacity; **(D)** or cisplatin; **(E)** and paclitaxel; **(F)** IC50 values.

In the panel of 19 ovarian cancer cell lines, *THBS1* gene expression (median 9.5; range = 4.8–13.2) was associated with *TP53* gene mutation status and protein expression. Specifically *THBS1* gene expression was higher in cells containing a wild-type *TP53* gene or null or frameshift *TP53* mutation compared to those harboring a missense *TP53* gene mutation (*p* = 0.005) (Table 1; Figure 1). *THBS1* gene expression was also higher in cells with low p53 protein expression (0 or 1+) compared to those exhibiting high p53 protein expression (2+) (*p* = 0.008) (Table 1). Pyrosequencing revealed a wide distribution of promoter methylation across the cell lines, but the majority showed low methylation levels (median = 8.6; range = 3.5–88.8). Cells with low levels of promoter methylation (≤5% methylated) exhibited higher *THBS1* gene expression (>5), while those with high levels of promoter methylation (>15% methylated) had low *THBS1* gene expression (<5) (Figure 2A). Since only three observations have *THBS1* gene expression values less than five the group is too small to use a statistical test for comparison. There was no association between *THBS1* promoter methylation and *TP53* gene mutation status (*p* = 1.0) or p53 protein expression (*p* = 1.0). All of the cells with promoter methylation >15% harbored a missense *TP53* gene mutation.

**Table 1:** **Relationship between *THBS1* differential gene expression, promoter methylation, mRNA expression, and *TP53* mutations in ovarian cancer cell lines**.

		*THBS1* promoter methylation percent	Wilcoxon rank sum	*THBS1* gene expression	Wilcoxon rank sum
	*n*	Median (IQR)	*p*	Median (IQR)	*p*
***TP53* MUTATION**					
Null	3	8.7 (8.0–9.4)	1.0*	9.9 (9.9–10.5)	0.005*
Wild type	5	8.9 (8.6–9.2)		10.1 (9.5–12.5)	
Missense	9	8.1 (7.8–47.1)		7.5 (5.1–7.9)	
Frame Deletion	1	8.0		9.6	
**TP53 PROTEIN OVEREXPRESSION**					
No: 0 or 1+	7	8.6 (7.8–10.0)	1.0	10.2 (8.9–12.2)	0.008
Yes: 2+	9	8.1 (7.8–47.1)		7.9 (5.1–9.4)	

*There were too few observations in each group for comparison testing. Therefore THBS1 gene expression was compared in the samples with missense mutations to those with null mutations and intact wild-type TP53 gene combined.

There was no association between *THBS1* gene expression and population doubling time of the cells, invasive capacity, anchorage-independent growth or cisplatin and paclitaxel IC50 values (Figures 2B–F).

**Figure 3:**
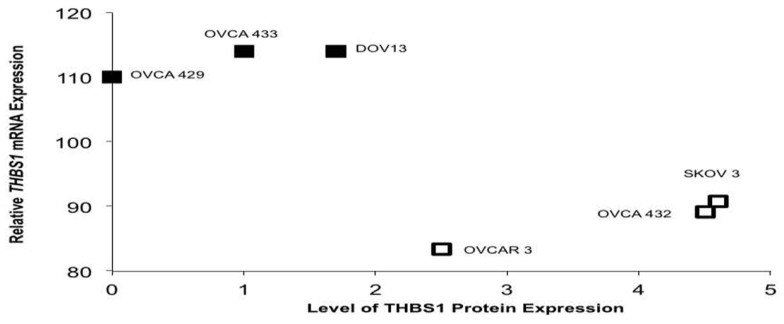
***TP53* mutation status in select ovariancancer cell lines and *THBS1* mRNA and protein expression**. The three ovarian cancer cell lines with wt *TP53* demonstrated higher levels of *THBS1* mRNA, but lower relative protein expression. Conversely, mutant TP53 cell lines had lower levels of *THBS1* mRNA expression and higher levels of relative protein expression. *TP53* missense mutation 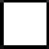; wt *TP53* gene 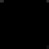.

We evaluated *THBS1* gene, mRNA, and protein expression in a subset of six ovarian cancer cell lines. Relative *THBS1* gene expression was higher in the wild-type cell lines (OVCA429, 13.6; DOV13, 12.4; OVCA433,14.0) compared to three cell lines harboring a *TP53* mutation (OVCAR 3, 7.6; SKOV3, 9.6; OVCA432, 9.5) (RMA-normalized values given are an average of Affymetrix U133 probes 201108_s_at, 201109_s_at, and 201110_s_at). We observed that the cells containing a wt*TP53* gene tended to express higher levels of *THBS1* mRNA (110–114), but lower relative protein expression (absent to 1.7). Conversely, mutant *TP53* cell lines had lower levels of *THBS1* mRNA expression (83–91) and higher levels of relative protein expression (2.5–4.6) (Figure 3).

#### The effect of radiation and hypoxia treatment on THBS1 mRNA expression in the A2780 ovarian cancer cell lines

**Figure 4:**
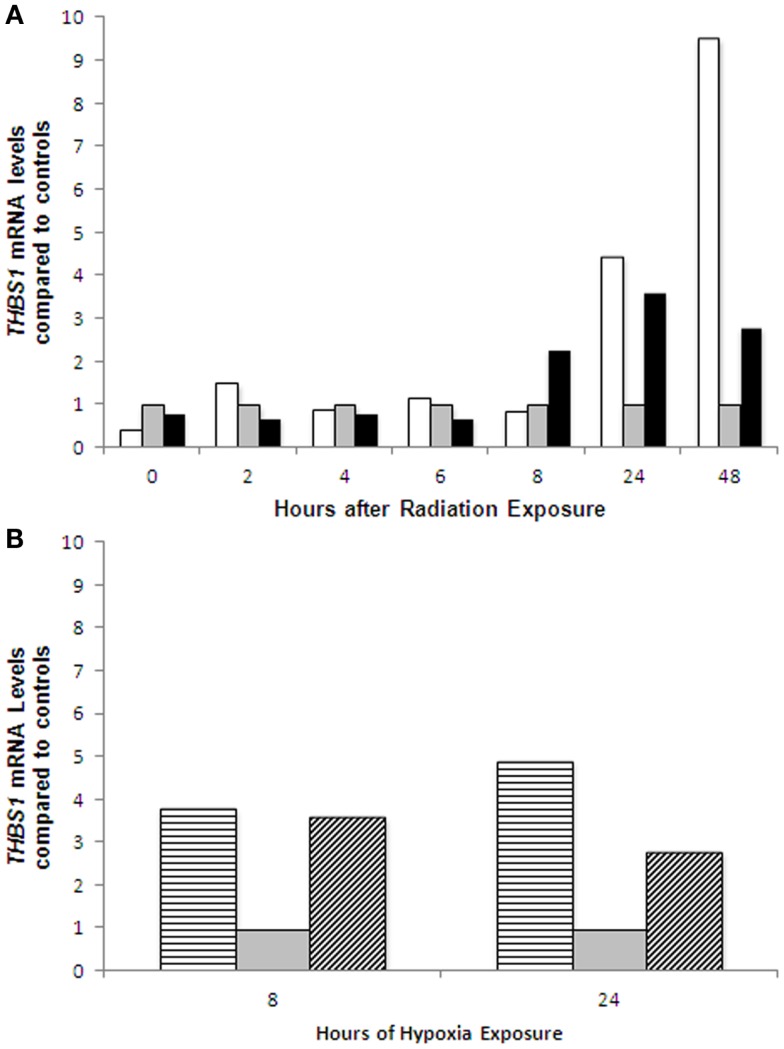
***THBS1* mRNA expression in ovarian cancer cell lines following radiation and hypoxia treatment**. Induction of *THBS1* transcription in the parent A2780wt*TP53* cells and A2780m*TP53* cells following radiation treatment **(A)**; and hypoxia exposure **(B)**. After treatment with radiation, the A2780wtTP53 cells demonstrated a 3.6-fold increase at 24 h while the A2780m*TP53* cells had a 4.5-fold increase at 24 h and a 9.5-fold increase at 48 h. There was a 3.4-fold greater increase in THBS1 levels at 48 h in the A2780m*TP53* cell line compared to wild type. There was an approximately fourfold increase in THBS1 levels in the A2780wt cells at 8 and 24 h. In the A2780m there was a 4.6-fold increase at 8 h, and a 2.8-fold increase at 24 h. Controls 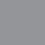; radiated A2780wt*TP53* cells 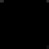; radiated A2780m*TP53* cells 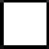; hypoxia treated A2780wt*TP53* cells 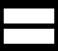; and hypoxia treated A2780m*TP53* cells 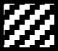.

**Figure 5:**
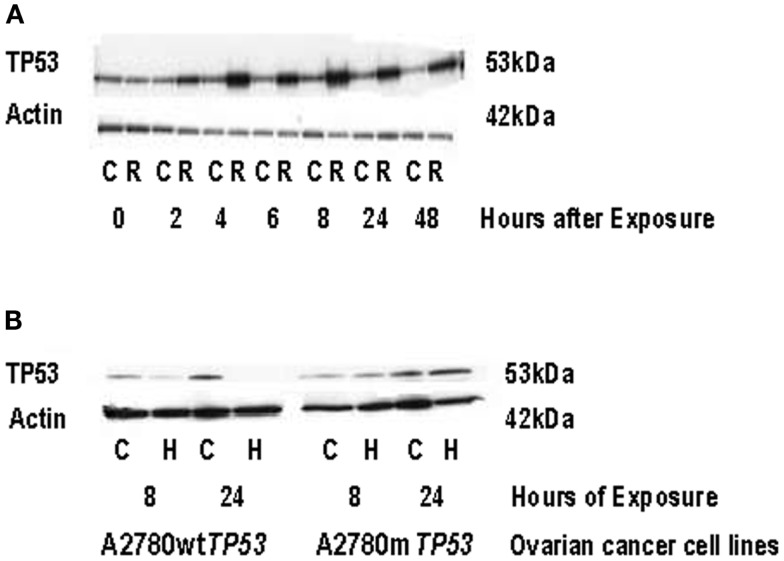
**TP53 protein expression in ovarian cancer cell lines following radiation and hypoxia treatment**. **(A)** Induction of TP53 protein expression in A2780wt*TP53* cells following radiation treatment. Irradiated cells demonstrated a 3.3-fold increase in TP53 expression at 48 h compared to non-irradiated cells. **(B)** Induction of p53 protein expression in the parent A2780wt*TP53* cells and A2780m*TP53* cells following hypoxia exposure. TP53 exposure was absent in the A2780wt*TP53* cells at 24 h of hypoxia exposure. There was no significant increase in p53 expression in the A2780m*TP53* cells after hypoxia. C, control; R, radiation; H, hypoxia.

After treatment with radiation, the A2780wt*TP53* cells demonstrated a 3.6-fold increase in *THBS1* mRNA levels at 24 h while the A2780m*TP53* cells had a 4.5-fold increase at 24 h and a 9.5-fold increase at 48 h (Figure 4A). There was a 3.4-fold greater increase in *THBS1* mRNA levels in the A2780m*TP53* cell line compared to wild-type (Figure 4A). Similarly, when compared to non-irradiated cells, irradiated cells demonstrated a 3.3-fold increase in p53 protein expression 48 h after exposure (Figure 5A).

Following exposure to hypoxia, the *THBS1* mRNA levels increased approximately fourfold in A2780wt*TP53* cells at 8 and 24 h. There was a similar increase in *THBS1* mRNA levels in the A2780m*TP53* cells with a 4.6-fold increase at 8 h and a 2.8-fold increase at 24 h (Figure 4B). In contrast, p53 expression was absent in the A2780wt*TP53* cells at 24 h and there was no significant increase in p53 expression in the A2780m*TP53* cells after exposure to hypoxia (Figure 5B).

## Discussion

The function of THBS1, its prognostic effect in various cancers, and its regulation are controversial. Dameron et al. previously reported that THBS1 may be regulated by *TP53* based on studies of fibroblasts from patients with Li Fraumeni syndrome (1). Our data from immortalized ovarian cell lines indicate that *THBS1* expression is associated with *TP53* status and is consistent with the hypothesis that *THBS1* gene expression is regulated via a *TP53*-dependent pathway. The ovarian cancer cell lines containing wt *TP53* expressed higher levels of *THBS1* mRNAs. In contrast, the cell lines harboring missense mutant *TP53* expressed low *THBS1* mRNA levels. Furthermore, induction with radiation, known to stimulate *TP53* transcription, was associated with a distinct increase in *THBS1* mRNA levels.

**Figure 6:**
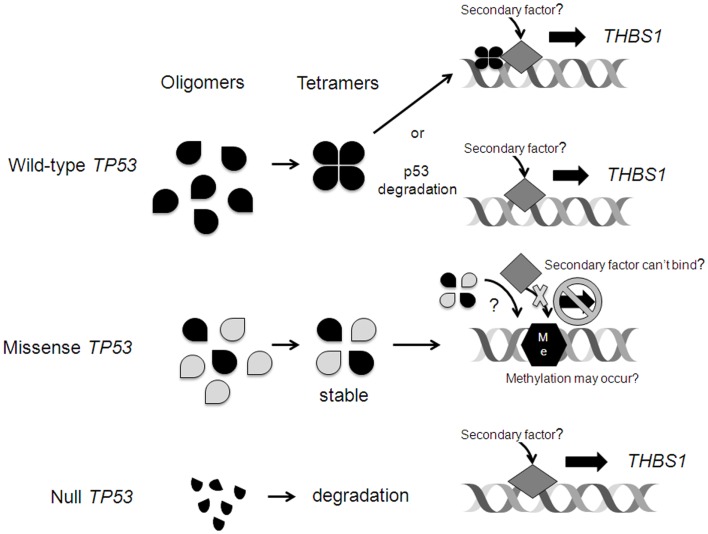
**Possible mechanisms of *TP53* regulation of *THBS1* expression**. Wild-type (wt) *TP53* may bind to the *THBS1* promoter resulting in gene transcription. Alternatively, wt*TP53* is normally degraded and expressed at low levels allowing for a secondary factor to bind to the promoter site. Missense p53 protein can bind with wt p53 protein and prevent it from forming homotetramers and/or interacting with DNA, or if it does interact with DNA, the presence of the mutant protein may impede interaction with other secondary factors required to drive induction of transcription. In rare cases, upon mutation the wt p53 DNA-binding activity is lost and the p53 target regions are vulnerable to *de novo* cytosine methylation (Me) that inhibits transcription. Null *TP53* mutations lead to complete loss of function of the gene and abnormal p53 fragments that are degraded. This may allow for a secondary factor to bind and induce *THBS1* transcription.

Wild-type *TP53* may bind to the *THBS1* promoter resulting in gene transcription. Alternatively, wt*TP53* is normally degraded and expressed at low levels, potentially allowing for a secondary factor to bind to the promoter site. Interestingly, radiation induced *THBS1* expression was most pronounced in the mutated *TP53* cell line, and we don’t have a clear explanation for this finding. The A2780 daughter cell line carries a missense *TP53* mutation. However, the effect of the mutation on p53 function and DNA-binding activity is unknown. In general, *TP53* mutations change the amino acid encoded within the affected codon, act in a “dominant negative” fashion and can neutralize the function of the normal intact *TP53* allele. Specifically, missense p53 protein can bind with wt p53 protein and prevent it from forming homotetramers and/or interacting with DNA, or if it does interact with DNA, the presence of the mutant protein may impede interaction with other secondary factors required to drive induction of transcription (Figure 6). Missense and null *TP53* mutations and proteins may have different effects on gene transcription. Interestingly, the cell lines with null *TP53* mutations (those that lead to complete loss of function of the gene, which can include some truncation mutations that prematurely terminate the polypeptide, some non-sense mutations that introduce a stop codon through point mutation and prematurely truncate the polypeptide, and some frameshift mutations resulting from insertion or deletion of non-multiple-of-three nucleotides within the coding sequence that shift the open reading frame, leading to premature truncation of the polypeptide), demonstrated similar levels of *THBS1* expression as the wt *TP53* cell lines. Our findings suggest that missense, but not null *TP53* mutations, may interfere with *THBS1* regulation.

It is unlikely that methylation plays a significant role in the regulation of *THBS1* gene expression, given the overall low levels of *THBS1* promoter methylation detected in the vast majority of the cell lines analyzed. However, we did find that all of the cells with higher levels of promoter methylation (>15% methylated) harbored a missense *TP53* gene mutation and had the lowest *THBS1* gene expression. These findings suggest that *THBS1* gene expression may be silenced in association with aberrant cytosine methylation of its promoter (Figure 6). Transcriptional repression of *MASPIN*, a tumor-suppressor gene involved in angiogenesis, and *desmocollin 3 (DSC3)*, an inhibitor of cell motility, by aberrant DNA methylation has been reported (10, 24). In these cases, the wt *TP53* gene binds to its consensus DNA-binding sites within the promoter, prevents aberrant *de novo* cytosine methylation therefore protecting the potential for gene activation. However, when the *TP53* gene is mutated, its DNA-binding activity is lost and the *TP53* target regions are vulnerable to methylation; thus the ability to activate transcription is repressed (10). Hypermethylation of the *THBS1* promoter in tumor specimens has been associated with worse clinical outcome in patients with neuroblastomas (25) and penile cancers (26) and an aggressive phenotype in those with gastric cancers (27). In melanoma cell lines, exposure to a demethylating agent reversed *THBS1* promoter hypermethylation, increased THBS1 expression, and reduced angiogenesis *in vivo* (28). However, Miyamoto et al. reported that *THBS1* methylation was more frequent in gastric cancers with wt *TP53* compared to those with mutant *TP53* (27). The association between *THBS1* promoter methylation and survival in patients with EOC has not yet been explored. However, our findings in ovarian cancer cell lines, which may or may not be representative of the situation in primary tumor tissues, indicate that *THBS1* promoter methylation is relatively low and that the regulation of *THBS1* is probably not primarily driven by differences in methylation, at least in these cells.

Hu and colleagues recently reported that *THBS1* promoter methylation was induced in the setting of oxygen-glucose deprivation (29). Oxygen–glucose deprivation-induced *THBS1* promoter methylation was associated with a reduction in *THBS1* mRNA and protein expression. According to Wang et al. glucose up-regulates *THBS1* gene transcription through antagonism of cGMP-dependent protein kinase repression via upstream stimulatory factor 2 (30). All of our experiments were conducted in glucose-based media that may have interfered with the methylation process. We also found that hypoxia induced *THBS1* mRNA expression, which was not expected. Furthermore, hypoxia exposure did not elicit an increase in p53 protein expression, suggesting that the increase in *THBS1* expression was not mediated by the *TP53* pathway. We hypothesized that hypoxia would lead to reduced *THBS1* expression to confer a favorable angiogenic environment. However, others have also reported that hypoxia increases THBS1 expression. Ortiz-Masia and colleagues found that hypoxia exposure resulted in hypoxia-inducible factor-1 (HIF1) dependent up-regulation of *THBS1* (31). HIF1 binds to the HRE sequence in the *THBS1* promoter. The HIF1 pathway represents another venue of THBS1 regulation. Other mechanisms of *THBS1* regulation may include epigenetic modulation via histone modifications, transcriptional repressors and enhancers that augment or inhibit binding and activity or regulation via other tumor-suppressor genes (30, 32, 33).

We also noted a paradoxical relationship between *THBS1* relative gene, mRNA, and protein expression. The ovarian cancer cell lines harboring mutant *TP53* genes had lower relative *THBS1* mRNA levels, but expressed higher THBS1 protein. In contrast, ovarian cancer cell lines with wt *TP53* had higher relative *THBS1* gene and mRNA levels, but expressed lower THBS1 protein levels. Sundaram and colleagues observed a similar paradoxical relationship in a colorectal cancer cell line (34). While *TP53* stimulated *THBS1* transcription, there was not an associated increase in THBS1 protein levels. They discovered that *TP53* upregulated a microRNA, miR-194, in THBS1 retrovirus-transduced HCT116 cells, leading to decreased THBS1 levels. The removal of the miR-194 complementary site in the *THBS1* 3′-untranslated region, led to *THBS1* reactivation, impaired angiogenesis in Matrigel plugs, and reduced growth of HCT116 xenografts. In contrast, transient overexpression of miR-194 increased angiogenesis in HCT116/THBS1 cells, and increased microvascular densities and vessel sizes *in vivo*. The findings indicate that miR-194 is involved in the post-transcriptional regulation of THBS1. However, their findings do differ from ours in that they also reported that *TP53* stimulated *THBS1* transcription did not increase with *THBS1* mRNA levels. Further analysis is needed to understand the *THBS1* post-transcriptional modifications in ovarian cancer.

Moreover, post-translational modifications may be critical in altering THBS1 protein expression (35). Thrombospondins are large trimeric polypeptides and known targets of proteolysis. The THBS1 and 2 activities are uniquely determined by exposure to the microenvironment. Proteolytic cleavage transforms their structure and alters their activity in a tissue- and pathophysiological-specific manner (35). THBS1 protein can be cleaved by cathepsins, leukocyte elastases, and plasmin. It was beyond the scope of our project to explore post-transcriptional or translational modifications to account for our findings.

Of note, we did not find an association between *THBS1* expression *in vitro* and tumor growth properties or cisplatin/paclitaxel induced growth inhibition. However, *THBS1*’s angiogenic effect and interaction with cytotoxic agents may not be adequately elucidated using *in vitro* studies. The *in vitro* nature of this investigation limits the application of these results and does not account for the role of the tumor microenvironment. Specifically cell lines are cancer cells only and the adjacent stroma that is integral to evaluate mesenchymal remodeling and tumor angiogenesis was not assessed in this model. Other limitations of our study included the use of a simplistic model of ionizing radiation to simulate *TP53* induction. To validate our model we irradiated ovarian cancer cell line A2780wt*TP53* to 5 Gy and then subjected cell lysates to immunoblot to assess total protein expression. When compared to non-irradiated cells, irradiated cells demonstrated a 3.3-fold increase in p53 expression 48 h after exposure. We acknowledge that ionizing radiation may also induce other genes in addition to *TP53* and these unidentified genes may also play a role in angiogenesis. We are continuing to evaluate *THBS1* expression and promoter methylation in ovarian cancer specimens that contain cancer cells as well as the surrounding stroma.

In conclusion, *THBS1* expression may be regulated via the *TP53* pathway and induced by hypoxic tumor microenvironment conditions. Overall low levels of *THBS1* promoter methylation imply that methylation is not the primary driver of *THBS1* expression in EOC. *THBS1* expression does not appear to be associated with tumor growth properties.

## Conflict of Interest Statement

The authors declare that the research was conducted in the absence of any commercial or financial relationships that could be construed as a potential conflict of interest.
